# Insights into Polymeric Materials for Prosthodontics and Dental Implantology

**DOI:** 10.3390/ma15155383

**Published:** 2022-08-04

**Authors:** Artak Heboyan, Muhammad Sohail Zafar, Mohmed Isaqali Karobari, João Paulo Mendes Tribst

**Affiliations:** 1 Department of Prosthodontics, Faculty of Stomatology, Yerevan State Medical University after Mkhitar Heratsi, Str. Koryun 2, Yerevan 0025, Armenia; 2 Department of Restorative Dentistry, College of Dentistry, Taibah University, Al Madinah Al Munawwarah, Medina 41311, Saudi Arabia; 3 Department of Dental Materials, Islamic International Dental College, Riphah International University, Islamabad 44000, Pakistan; 4 Department of Conservative Dentistry & Endodontics, Saveetha Dental College & Hospitals, Saveetha Institute of Medical, Technical Sciences University, Chennai 600077, India; 5 Department of Restorative Dentistry & Endodontics, Faculty of Dentistry, University of Puthisastra, Phnom Penh 12211, Cambodia; 6 Department of Dental Materials, Academic Centre for Dentistry Amsterdam (ACTA), University of Amsterdam and Vrije Universiteit Amsterdam, 1081 LA Amsterdam, The Netherlands

Nowadays, a large variety of prostheses both in medicine and dentistry are increasingly made of polymers and polymer-based materials [1,2,3,4,5,6,7,8,9,10,11,12]. The physical characteristics of Poly(methyl methacrylate) (PMMA) such as durability, strength, light weight and high impact toughness prevail over those of glass and polystyrene, while its resistance to the environment is much higher as compared to other plastics indicated for biomedical application [1,2,3]. However, PMMA in several chemicals and organic solvents can easily hydrolyze esters, resulting in soak and decomposition. This represents its major drawback [1,2]. Therefore, new studies evaluating the reduction of biofilm formation on prosthetic devices, particularly on denture base, is a goal of crucial importance for overall dental oral health.

In order to minimize the colonization of bacteria and fungi on PMMA, several active nanoparticles can be incorporated on its structure prior to the final polymerization process. Zirconium dioxide nanoparticles (ZrO_2_-NPs), silver (Ag-NPs), or platinum nanoparticles (Pt-NPs) are among the innumerous particles that are currently being incorporated in PMMA [2]. Silver nanoparticles can improve its heat conduction, providing a better comfort to patients [2]. In addition to its mechanical properties’ improvement, the usage of ZrO_2_ nanoparticles decrease the biofilm adherence to denture bases and removable dentures. Depending on the dose, the *C. albicans* biofilm’s bioactivity can be properly reduced, showing anti-adhesion activity when a high Ag-NP concentration (5%) is used [3,5,6].

In addition to the use of nanoparticles, the prevention of biofilm formation can be achieved via the manufacturing of different polymeric nanofilms to cover biomedical devices. Phospholipid polymer, for example, can be used to prevent *S. mutans* adherence on a denture base [10]. As shown by the results of in vitro studies, nanocomposites can be effective in destroying both Gram-positive and Gram-negative strains, while it does not produce significant cytotoxic effects on osteoblast an fibroblasts-like cells or adipose tissue-derived stem cells [6,12].

Another alternative to improve the polymers’ properties is an associating of different polymers with promising structures. Defined as the “thinnest material in the universe”, graphene is arranged in a honeycomb crystal grid and is currently present in innumerous spheres of science, as well as in dentistry, due to its striking mechanical characteristics. Studies have been carried out on the technology of manufacturing graphene sheets with better characteristics and applicability [13]. Properties of graphene oxide biomaterials are of great interest for dentistry with the aim to manufacture different nanocomposites of different polymer matrices for further application in innumerous dental specialties. It is also possible to lessen graphene and make it function with other polymeric materials in order to fabricate antimicrobial nanocomposites [14]. Therefore, further studies with graphene associated with dental polymers have high potential to be used as references for the production of antimicrobial surface of dental prosthesis and other biomaterials.

Despite the success of classical PMMA and resin composites, high-density polymers are increasingly becoming popular in the field of dentistry. The so-called PEEK (polyether ether ketone) from the polyaryletherketone (PAEK) family is a polymeric biomaterial used to replace metals and weaker polymers in both fixed and removable prosthetic devices. The modulus of elasticity of the PEEK is lower (≈4 GPa) compared with the ceramics and metals (≈ 60–200 GPa), which provides an alternative for better dissipation of functional chewing loads. Moreover, PEEK can serve as a substitute to conventional superstructures and denture base resin biomaterials with enhanced mechanical and lower discoloration properties. Additionally, composites and titanium dioxide can be added for the improvement of its esthetic characteristics. PEEK also demonstrates ideal chemical resistance. Therefore, it can tolerate high temperatures without any substantial degradation. Due to its exceptionally low allergenic potential, it can rarely induce immune responses after intraoral application [5,7,8,9,10]. Therefore, further studies evaluating this property can contribute to the scientific literature and truly demonstrate the potential usage and disadvantages of this property for prosthetic and implant dentistry.

Although the application of various polymeric biomaterials in prosthodontics and dental implantology has been increased (due to their acceptable mechanical and biological characteristic) to make the chewing loads stand out, there are several fields of knowledge that still require improvements for reliable clinical applications [11,12,13,14,15]. One of the hot topics in accelerated development is the additive manufacturing technique, which allows the dentist to obtain complex restorations and structures with a high precision based on a virtual designed model. For each indication, there are different 3D-printers and polymers available, such as polycarbonates, polylactic acid, polyurethanes, polyether ketone ketone, polylactic-co-glycolic acid, polyether ether ketone, polyvinyl alcohol, polyurethane, polycaprolactone, poly-glycolic acid, polybutylene terephthalate, acrylonitrile butadiene styrene, polymethyl methacrylate, etc. [2,3,7]. Therefore, the scientific literature can benefit from new information about them in terms of processing, disinfection, dimensional stability, and the proper method of waste disposal from the subproducts.

Implant osseointegration requires appropriate conditions. Low vasculature at the implant/bone interface due to insufficient oxygen concentration leads to the electrons accumulation which might promote infection and inflammatory process, ending in the implant’s rejection. In the case of high-resistance fiber-reinforced fillers and complex additives, or antimicrobial combination by means of thermoset polymers, curing at low temperatures can be used to modify their surface. This ensures a better condition for osseointegration and assists in managing the issues in contact with the dental implants’ surface [16].

The current goal of researchers is to develop dental implant bioactive coatings to strengthen osseointegration through interaction between the protein-cell-tissue and the surface of the implant. Using an appropriate coated material, bone-resorbing medications applied locally in the tissues around implants greatly contribute to successful dental implant treatment [15,16,17,18,19]. These bioactive coatings have been reported to provide better initial cell adhesion. Other nanofiber-based polymeric coatings containing antibiotic can also be applied on implants to decrease the risk of implant loss (especially in patients with chronic periodontitis). Meanwhile, long-term antimicrobial medication releases, such as metronidazole, ciprofloxacin, or minocycline, were supported by polymeric coatings [7,20]. Nevertheless, some mechanical properties of polymeric coatings can be low and its use can be compromised due to plastic deformation at high stress levels [5]. In this sense, more studies in this topic are needed.

Finally, this editorial invites readers to search for and share further studies linked with the development of dental procedures and biomaterials for oral and maxillofacial reconstructive treatments which can be improved with polymeric materials.
